# A role for TRPC3 in mammalian testis development

**DOI:** 10.3389/fcell.2024.1337714

**Published:** 2024-02-15

**Authors:** Zhenhua Ming, Stefan Bagheri-Fam, Emily R. Frost, Janelle M. Ryan, Brittany Vining, Vincent R. Harley

**Affiliations:** ^1^ Sex Development Laboratory, Hudson Institute of Medical Research, Melbourne, VIC, Australia; ^2^ Department of Molecular and Translational Science, Monash University, Melbourne, VIC, Australia

**Keywords:** SOX9, testis, sertoli cells, DSD, TRPC3, TRP, sex determination

## Abstract

SOX9 is a key transcription factor for testis determination and development. Mutations in and around the *SOX9* gene contribute to Differences/Disorders of Sex Development (DSD). However, a substantial proportion of DSD patients lack a definitive genetic diagnosis. SOX9 target genes are potentially DSD-causative genes, yet only a limited subset of these genes has been investigated during testis development. We hypothesize that SOX9 target genes play an integral role in testis development and could potentially be causative genes in DSD. In this study, we describe a novel testicular target gene of SOX9, *Trpc3*. *Trpc3* exhibits high expression levels in the SOX9-expressing male Sertoli cells compared to female granulosa cells in mouse fetal gonads between embryonic day 11.5 (E11.5) and E13.5. In XY *Sox9* knockout gonads, *Trpc3* expression is markedly downregulated. Moreover, culture of E11.5 XY mouse gonads with TRPC3 inhibitor Pyr3 resulted in decreased germ cell numbers caused by reduced germ cell proliferation. *Trpc3* is also expressed in endothelial cells and Pyr3-treated E11.5 XY mouse gonads showed a loss of the coelomic blood vessel due to increased apoptosis of endothelial cells. In the human testicular cell line NT2/D1, TRPC3 promotes cell proliferation and controls cell morphology, as observed by xCELLigence and HoloMonitor real-time analysis. In summary, our study suggests that SOX9 positively regulates *Trpc3* in mouse testes and TRPC3 may mediate SOX9 function during Sertoli, germ and endothelial cell development.

## 1 Introduction

Differences/Disorders of Sex Development (DSDs) are a heterogenous group of congenital conditions in which the development of chromosomal, gonadal and/or anatomic sex is atypical (Hughes et al., 2006). A notable subset of DSDs is 46, XY DSD, where individuals possess a 46, XY chromosome complement but present with atypical male or even female external genitalia, with or without Müllerian duct derived structures (female internal genitalia) (Wisniewski et al., 2019; Reyes et al., 2023). 46, XY DSD is associated with genetic mutations in key genes involved in testis development, such as the testis-determining gene *SRY*, *SOX9*, *NR5A1*, *MAP3K1*, and *DHX37* (Eggers et al., 2016; Xu et al., 2019; Reyes et al., 2023). Despite advancements in genomic technologies, many 46, XY DSD patients lack a clinical genetic diagnosis, and numerous genes contributing to DSD remain unidentified. It is therefore important to define the basic mechanisms underlying testis development and identify genes downstream of SOX9 that mediate its functions and potentially contribute to DSD.

Mammalian testes originate from bipotential gonads, where SRY expression commences at embryonic day 11.5 (E11.5) in mice (equivalent to 7 weeks gestation in humans), initiating the male sex differentiation pathway (Koopman et al., 1990; Koopman et al., 1991). In XY mouse gonads, SRY activates the pivotal testis gene *Sox9* in supporting cells, leading to their differentiation into Sertoli cells (Vidal et al., 2001; Chaboissier et al., 2004; Barrionuevo et al., 2006; Sekido and Lovell-Badge, 2008). Sertoli cells then proliferate, migrate and surround germ cells to form testis cords. Concurrently, Leydig cells outside the testis cords begin differentiation and produce androgens, which drive the development of male internal and external genitalia (Svingen and Koopman, 2013). In the female mouse XX gonad, ovarian development is also initiated at E11.5 through RSPO1-WNT4 and FOXL2 signaling pathways (Ottolenghi et al., 2007; Chassot et al., 2008). Within the developing testis, SOX9 plays a central role in regulating target genes, directing them in fulfilling their specific functions. For instance, SOX9 directly regulates the *Amh* gene, leading to the regression of Müllerian ducts in males (Arango et al., 1999; Barrionuevo et al., 2006). SOX9 also directly regulates the expression of *Dhh* to drive Leydig cell differentiation and *Cyp26b1* to inhibit the entry of XY germ cells into meiosis (Barrionuevo et al., 2006; Kashimada et al., 2011; Rahmoun et al., 2017). Given the pivotal role of SOX9 in testis development, our previous study explored the genes affected by the absence of *Sox9* and those bound by SOX9 at the post sex determination stage (Rahmoun et al., 2017). Among these candidate target genes of SOX9, *Trpc3* (transient receptor potential cation channel subfamily C member 3) stands out prominently due to its increased expression in Sertoli cells shortly after the onset of *Sox9* transcription (Jameson et al., 2012).

TRPC3 is a membrane protein that forms a non-selective calcium permeant cation channel. It is directly activated by diacylglycerols (DAG) in response to receptor-phospholipase C (PLC) pathways (Hofmann et al., 1999; Li et al., 1999). The structure of TRPC3 includes six transmembrane domains (TM1-TM6), and cytoplasmic N- and C-termini. The C-terminus includes a highly conserved transient receptor potential (TRP) domain and a calmodulin/inositol 1,4,5-trisphosphate (IP3) receptor-binding (CIRB) region (Liu et al., 2020). TRPC3 is abundantly expressed in the cerebellum, cerebrum and the cardiovascular system with essential roles in the regulation of neurogenesis and calcium signaling (Li et al., 1999; Gonzalez-Cobos and Trebak, 2010). Dysfunctions in TRPC3 are associated with neurodegenerative diseases, cardiac hypertrophy, and ovarian cancer (Yang et al., 2009; Becker et al., 2011; Kitajima et al., 2016). TRPC3 is found in both mouse and human sperm (Treviño et al., 2001; Castellano et al., 2003) and Pyr3, a TRPC3 antagonist, can inhibit mouse sperm motility and accelerate capacitation-associated protein tyrosine phosphorylation (Ru et al., 2015). However, the role of TRPC3 in testis development remains unclear.

In this study, we analyzed *Trpc3* expression and its role in XY and XX gonads during gonad development. The *Trpc3*/TRPC3 gene and protein showed strong expression in the SOX9-expressing Sertoli cells of the fetal XY gonad. Inhibition of TRPC3 in XY gonad culture leads to a decrease in germ cell numbers attributed to reduced germ cell proliferation. This inhibition also disrupts the coelomic blood vessel and increases endothelial cell apoptosis during testicular development. *In vitro* analysis further demonstrates that TRPC3 stimulates Sertoli cell proliferation and regulates cell morphology. Our findings emphasize the necessity of SOX9 for *Trpc3* expression in Sertoli cells and suggest that TRPC3 contributes to testis development by influencing the development of Sertoli, germ, and endothelial cells.

## 2 Materials and methods

### 2.1 Mice

All animal experimentation was approved and conducted in accordance with the guidelines established by the Monash Medical Centre Animal Ethics Committee. The generation of *AMH-Cre;Sox9* ^*flox/flox*^ mice was previously described (Barrionuevo et al., 2009), and these mice were maintained on a C57BL/6 background. Embryos were collected at E13.5, with the morning of plug identification designated as E0.5. Genotyping primers and PCR conditions were used as previously reported (Kist et al., 2002; Lécureuil et al., 2002; Jiménez et al., 2003).

### 2.2 Quantitative RT-PCR

Total RNA was extracted from fetal gonads (with mesonephros removed) at E13.5 or NT2/D1 cells using the RNeasy Mini kit (Qiagen). cDNA was synthesized using the QuantiTect reverse transcription kit (Qiagen), and quantitative PCR was performed using the QuantiNova SYBR green PCR kit (Qiagen) on a QuantStudio™ 6 Flex Real-Time PCR System (Applied Biosystems). Mouse primer sequences were as follows: *Trpc3* F: 5′TTA​TCG​ACT​ACC​CCA​AGC​AA3′, *Trpc3* R: 5′CCA​CAT​CAT​CCC​AAG​AAC​C3′, *Tbp* F: 5′ACG​GAC​AAC​TGC​GTT​GAT​TTT3′, *Tbp* R: 5′ACT​TAG​CTG​GGA​AGC​CCA​AC3’. Human primer sequences were as follows: *TRPC3* F: 5′TGC​TGC​TTT​TAC​CAC​TGT​AG3′, *TRPC3* R: 5′GTT​GAG​TAA​AAC​GAC​CAC​CA3′, *GAPDH* F: 5′GGA​GTC​AAC​GGA​TTT​GGT​C3′, *GAPDH* R: 5′TCC​ATT​GAT​GAC​AAG​CTT​CC3’. Relative expression was calculated using the delta delta cycle threshold (ΔΔCt) method with *Tbp* or *GAPDH* as the normalizing control. Statistical significance was determined using one-way ANOVA, followed by the Dunnett multiple comparisons test or unpaired Student’s t*-*test performed in GraphPad Prism 8.

### 2.3 *Ex vivo* gonad culture

Gonads with attached mesonephros were dissected from E11.5 Swiss mouse embryos. Genotyping analysis for sexing was performed using genomic DNA isolated from tail tissue, as previously described (Jiménez et al., 2003). Pairs of gonads from each embryo were cultured in hanging droplets of DMEM medium (Gibco) supplemented with 10% FBS (Bovogen) and 1% antibiotic-antimycotic (Gibco) (Ryan et al., 2011; Bernard et al., 2012). The optimal concentration of the TRPC3 inhibitor Pyr3 for gonad culture was determined based on previous studies demonstrating that 3 µM of Pyr3 induces maximal inhibition of TRPC3-mediated Ca^2+^ influx (Kiyonaka et al., 2009; Kim et al., 2011). Our MTS assay confirmed that 3 µM Pyr3 did not adversely affect cell viability in NT2/D1 cells (data not shown). Therefore, gonads were treated with 3 µM of the TRPC3 inhibitor Pyr3 (Sigma Aldrich) or an equivalent volume of the vehicle DMSO. The explants were cultured in a humidified cell culture incubator set at 37°C with 5% CO_2_. After 24 h, the drug treatment was washed off, fresh medium was replaced, and the culture was continued for either 24 or 48 h. The gonads were then harvested and fixed in 10% neutral buffered formalin for subsequent immunofluorescence staining.

### 2.4 Immunofluorescence

The fixed samples were processed and embedded in paraffin. Paraffin blocks were sectioned at 4 μm and mounted onto slides. Immunofluorescence analysis was conducted as previously described (Bagheri-Fam et al., 2017). Primary antibodies used were anti-TRPC3 rabbit polyclonal (ab51560, 1:50, 20 μg/mL, Abcam), anti-AMH mouse monoclonal (sc-166752, 1:100, 2 μg/mL, Santa Cruz), anti-SOX9 rabbit polyclonal (AB5535, 1:1000, 1 μg/mL, Merck), anti-DDX4 goat polyclonal (AF 2030, 1:500, 0.4 μg/mL, R&D Systems), anti-Laminin rabbit polyclonal (L9393, 1:200, 2.5 μg/mL, Merck), anti-GATA4 mouse monoclonal (sc-25310, 1:100, 2 μg/mL, Santa Cruz), anti-PECAM1 goat polyclonal (AF3628, 1:100, 2 μg/mL, R&D Systems), anti-PH3 rabbit polyclonal (06570, 1:1000, 1 μg/mL, Merck), anti-HSD3B mouse monoclonal (sc-515120, 1:100, 2 μg/mL, Santa Cruz), and anti-cleaved Caspase-3 rabbit polyclonal (9661, 1:1000, 0.052 μg/mL, Cell Signaling). The secondary antibodies used were donkey anti-rabbit Alexa Fluor 488 (A21206), donkey anti-mouse Alexa Fluor 594 (A32744), and donkey anti-goat Alexa Fluor 647 (A32849) from Thermo Fisher Scientific (1:1000, 2 μg/mL). Slides were mounted and imaged using fluorescence microscopy or confocal microscopy (Olympus Corp).

### 2.5 xCELLigence

The human testicular cell line NT2/D1 cells were grown in DMEM/F12 GlutaMAX medium (Gibco) supplemented with 10% FBS (Bovogen) and 1% antibiotic-antimycotic (Gibco). Culture medium was replaced every two to 3 days, and cells were subcultured at a ratio of 1:2 to 1:3 using 0.05% trypsin-EDTA (Gibco) when reaching 90%–95% confluency. NT2/D1 cells were cultured in the serum-free starvation medium for 6 h before being seeded in E-plates (Agilent Technologies) at a density of 1×10^4^ cells per well. TRPC3 inhibitor Pyr3 was added at the start of the experiment. Readings were taken on the xCELLigence machine every minute for 72 h. Adhesion was analysed in the first 5 h, and proliferation was analysed in 5–72 h. All treatments were performed in quadruplicate to a total of three biological replicates. Rates of change were represented as slope (1/hr). Statistical significance was determined using one-way ANOVA followed by the Dunnett multiple comparisons test performed in GraphPad Prism 8.

### 2.6 HoloMonitor

The digital holographic microscope HoloMonitor (Phase Holographic Imaging AB) was used for real-time monitoring of cell morphology in culture. NT2/D1 cells were transfected with the siTRPC3 (SASI_Hs01_00195103, Merck) or the negative control siRNA (SIC001, Merck) using Lipofectamine RNAiMAX (13778–150, Invitrogen) according to the manufacturer’s instructions. After 48 h, cells were seeded into a 24-well lumox^®^ plate (94.6110.024, Sarstedt) at 5.073×10^4^ cells per well in 1.8 mL cell culture medium. After 5 h of cell adhesion, the standard plate lid was replaced with the HoloLids (71130, Phase Holographic Imaging AB). The plate was immediately placed on the stage of the HoloMonitor M4, which was kept inside a humidified incubator maintained at 37°C and 5% CO₂. HStudio 2.7 software was launched and set up according to the manufacturer’s manual. Multiple cellular parameters including cell count, confluence, cell area, optical thickness, volume, irregularity, and eccentricity were analysed. Two independent experiments were conducted for each experimental group, with phase contrast and holographic images being recorded from three randomly selected areas in each experiment.

## 3 Results

### 3.1 TRPC3 is strongly expressed in Sertoli cells from E13.5

**FIGURE 1:**
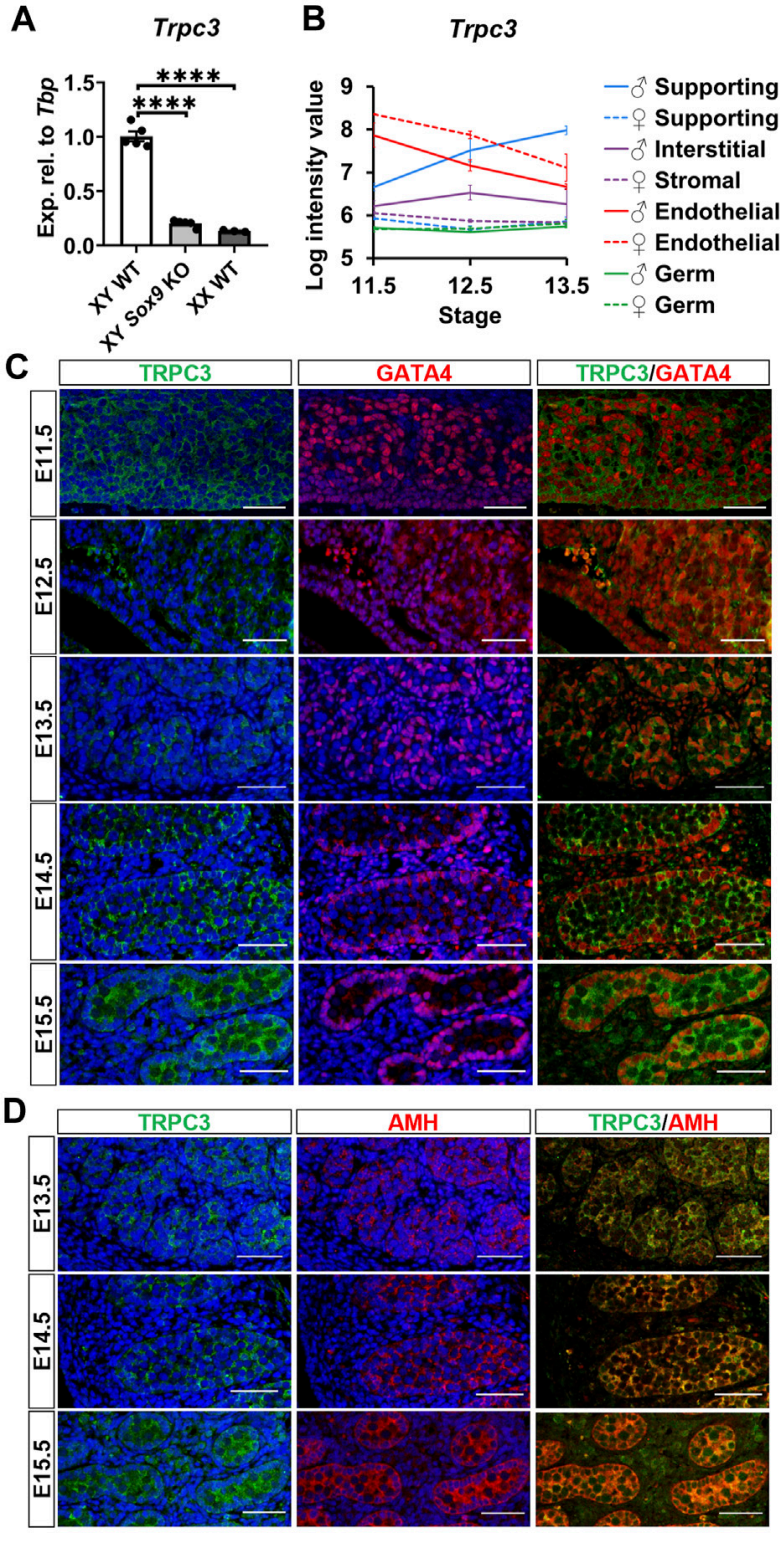
Expression of *Trpc3*/TRPC3 in the developing mouse testis. **(A)** qRT-PCR analysis confirming the downregulation of *Trpc3* in E13.5 mouse *Sox9* knockout gonads. n = 5 for XY wildtype (XY WT) and XY *Sox9* conditional knockout (XY *Sox9* KO), *n* = 3 for XX wildtype (XX WT). Transcript expression level of the *Trpc3* gene was normalized to *Tbp* and presented as relative expression. Mean ± SEM. One-way ANOVA test. ****p < 0.0001. **(B)** *Trpc3* RNA expression levels in developing male and female gonads from E11.5 to E13.5. The figure is generated based on the microarray dataset from Jameson et al. (2012). **(C, D)** Co-immunofluorescence staining of TRPC3 (green) with somatic cell nuclear marker GATA4 (red) and Sertoli cell cytoplasmic marker AMH (red) in the mouse testis from E11.5 to E15.5. Nuclei are visualized with the nuclear marker DAPI (blue). Scale bar = 50 μm.

We validated the downregulation of *Trpc3* in *AMH-Cre;Sox9* ^*flox/flox*^ (*Sox9* KO) testes at E13.5, in accordance with our previous RNA-sequencing data in *Sox9* KO testes (Rahmoun et al., 2017). qRT-PCR analysis revealed an 80% reduction of *Trpc3* expression in XY *Sox9* KO testes compared to XY wildtype (WT) testes at E13.5 (Figure 1A), with *Trpc3* expression levels close to those of XX WT gonads which were 86.7% lower than XY WT. We further investigated *Trpc3* expression during testis development by screening previous microarray data of male and female developing gonads from E11.5 to E13.5 (Jameson et al., 2012) and querying expression of *Trpc3* in isolated cell lineages of the developing male or female mouse gonad (Supplementary Figure S1). The expression of *Trpc3* increases in Sertoli cells between E11.5 and E13.5, while it remains absent from female supporting cells, stromal cells, and both male and female germ cells (Figure 1B). Taken together, these data suggest *Trpc3* expression is dependent on SOX9 during sex differentiation and is minimally expressed in XX gonads at E13.5.

We further assessed TRPC3 expression and localization in the developing male gonad between E11.5 and E15.5 through co-immunofluorescence staining together with either the somatic cell marker GATA4, which is strongly expressed in the nucleus of Sertoli cells, or with the Sertoli cell cytoplasmic marker AMH. TRPC3 protein was expressed at all five time points examined (Figure 1C). At E11.5 and E12.5, TRPC3 expression was identified in somatic cells. From E13.5 onwards, TRPC3 protein was clearly expressed in the Sertoli cell membrane and cytoplasm, as indicated by its colocalization with AMH (Figure 1D). Since the *Trpc3* gene is expressed in endothelial cells between E11.5 and E13.5 (Figure 1B), we also performed co-immunostaining for TRPC3 and the endothelial cell and germ cell marker PECAM1 between E11.5 and E15.5. At E12.5, E13.5 and E15.5, weak TRPC3 staining was found in a subset of endothelial cells of the coelomic blood vessel (Supplementary Figure S2). From E14.5 onwards, weak TRPC3 staining was also detectable in interstitial cells (Figures 1C, D). Co-immunostaining for TRPC3 and the Leydig cell marker HSD3B revealed that these are Leydig cells (Supplementary Figure S2). However, since these signals were also detected in the PECAM1 channel, it is possible that they represent nonspecific background staining (Supplementary Figure S2).

### 3.2 Inhibition of TRPC3 leads to a reduction in germ cell numbers in cultured XY gonads

**FIGURE 2:**
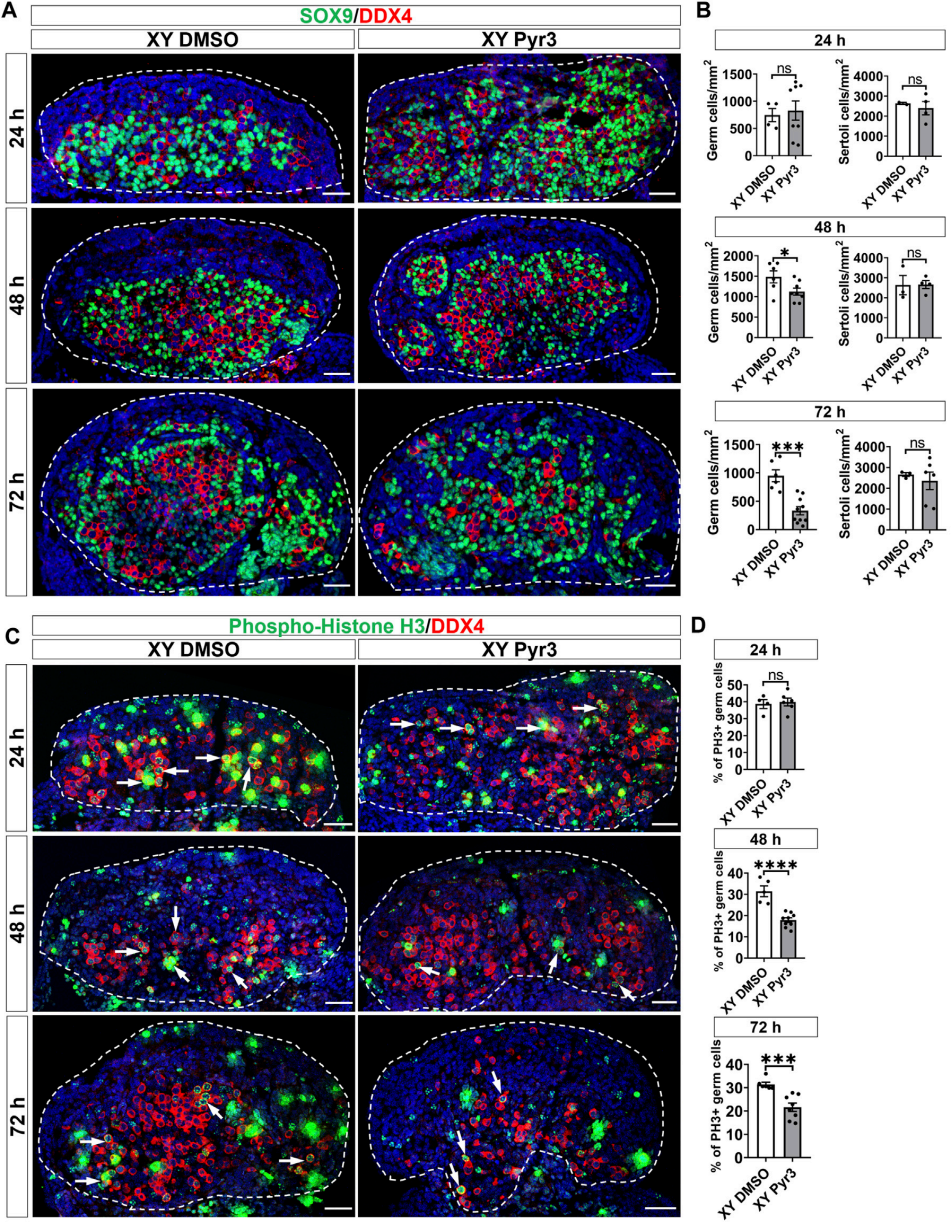
TRPC3 inhibition leads to a reduction in germ cell numbers and proliferation in cultured XY mouse gonads. **(A)** Immunofluorescence staining of male gonads after exposure to either the vehicle control DMSO or the TRPC3 inhibitor Pyr3 during *ex vivo* culture for 24, 48, and 72 h. The sections are stained for germ cell marker DDX4 (red) and Sertoli cell marker SOX9 (green). Nuclei are visualized with the nuclear marker DAPI (blue). Dashed lines outline gonads. Scale bar = 50 μm. **(B)** Quantification of germ cells and Sertoli cells in XY DMSO and XY Pyr3-treated gonads at 24-, 48- and 72-h post-culture. *n* = 2–4; sections counted = 2–10. Mean ± SEM. Unpaired Student’s t*-*test. *p < 0.05, ***p < 0.001; ns, not significant. **(C)** Immunofluorescence staining for the germ cell marker DDX4 (red) and cell proliferation marker phospho-histone H3 (PH3) (green). White arrows indicate examples of proliferating germ cells. **(D)** Quantification of proliferating germ cells is performed for PH3+ germ cells relative to the total germ cell population. n = 2–4; sections counted = 4–9. Mean ± SEM. Unpaired Student’s t*-*test. ***p < 0.001, ****p < 0.0001; ns, not significant.

To elucidate the role of TRPC3 in gonad development, we conducted *ex vivo* gonad culture experiments in which E11.5 male and female mouse gonads were treated with the TRPC3-specific inhibitor Pyr3 for 24 h, followed by 24 and 48 h culture in fresh media. Following the culture, gonads were analyzed by immunofluorescence for cell-specific markers such as SOX9 for Sertoli cells, DDX4 for germ cells, and GATA4 for somatic cells. After 72 h of culture, Pyr3-treated XY gonads exhibited a substantial reduction in germ cell numbers (2.9-fold) when compared to control XY gonads (Figures 2A, B). At the 48-h time point, Pyr3-treated XY gonads already exhibited a reduction in germ cell numbers (1.3-fold) in comparison to control XY gonads (Figures 2A, B), whereas after 24 h, germ cell numbers were still similar in Pyr3-treated and control XY gonads (Figures 2A, B). Distinct from the male gonads, TRPC3 inhibition did not cause any changes in germ cell numbers in female gonads (Supplementary Figure S3), highlighting a sex-specific response to TRPC3 inhibition.

To elucidate the mechanisms contributing to the reduced number of germ cells in Pyr3-treated XY gonads, we examined cell proliferation and apoptosis through immunofluorescence analysis in cultured gonads, using phospho-histone H3 (PH3) for proliferation assessment and cleaved Caspase-3 (CC3) for apoptosis. After 72 h of culture, the proportion of proliferating germ cells in control XY gonads was 31.44%, whereas Pyr3-treated XY gonads exhibited a reduced percentage of 17.84% (1.76-fold reduction) (Figures 2C, D). At the 48-h time point, Pyr3-treated XY gonads already displayed a significant 1.46-fold decrease in germ cell proliferation compared to XY control gonads (31.42% *versus* 21.58%). Notably, after 24 h of culture, the percentage of proliferating germ cells between the two groups was still comparable (38.64% *versus* 39.86%). Analysis of germ cell apoptosis revealed no significant differences between XY control gonads and XY Pyr3-treated gonads at the 24, 48 and 72-h time points (Supplementary Figure S4). Collectively, these findings suggest that the reduction of the germ cell population following TRPC3 inhibition is caused by decreased germ cell proliferation. Immunofluorescence analysis for Laminin, a marker for the basal lamina of testis cords, revealed that after 48 and 72 h of culture, both control and Pyr3-treated XY gonads had formed well-defined testis cords, wherein Laminin and a closely associated layer of Sertoli cells encircled both germ cells and Sertoli cells (Supplementary Figure S5).

### 3.3 Inhibition of TRPC3 perturbs the coelomic blood vessel in cultured XY gonads

**FIGURE 3:**
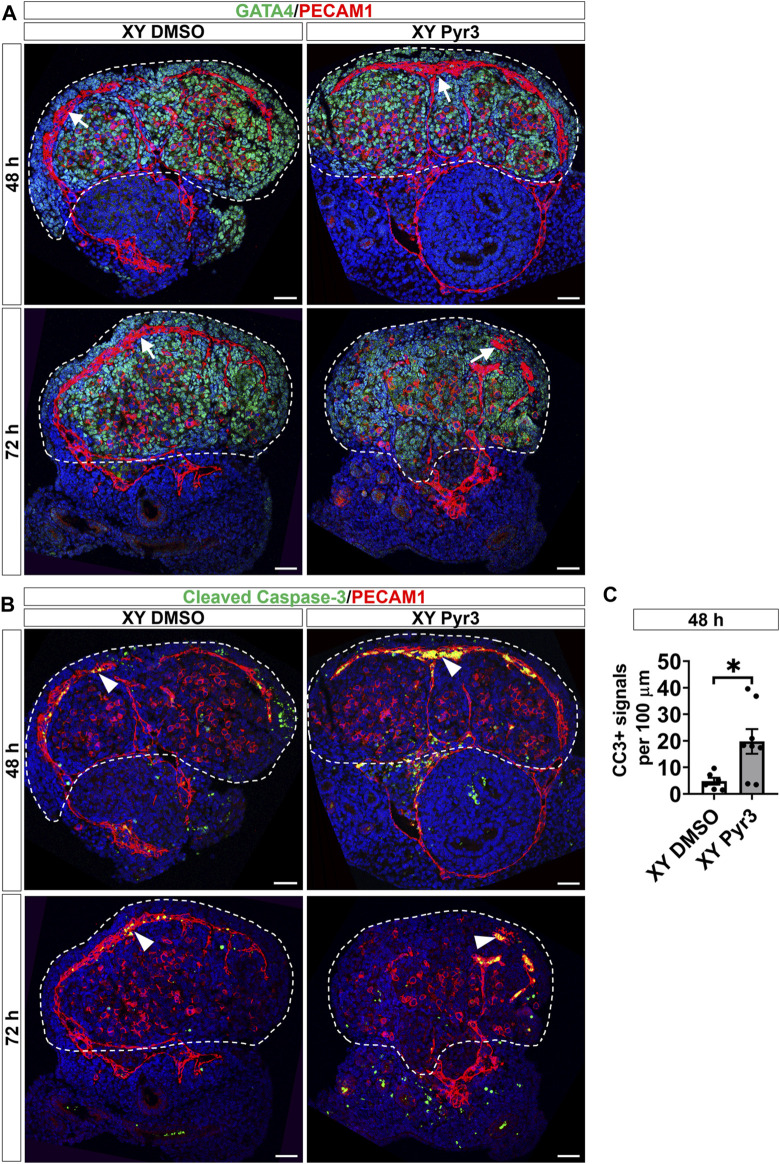
TRPC3 inhibition disrupts the coelomic blood vessel after 72 h of culture. **(A)** Immunofluorescence staining of GATA4 (green) and PECAM1 (red) in control (XY DMSO) and Pyr3-treated (XY Pyr3) gonads cultured *ex vivo* for 48 and 72 h. White arrows indicate the coelomic blood vessel. **(B)** Immunofluorescence staining of the cell apoptosis marker cleaved Caspase-3 (CC3) (green) and PECAM1 (red) in XY DMSO and XY Pyr3 gonads cultured *ex vivo* for 48 and 72 h. Arrowheads indicate examples of apoptotic endothelial cells. Nuclei are visualized with the nuclear marker DAPI (blue). Dashed lines outline gonads. Scale bar = 50 μm. **(C)** Quantification of apoptotic endothelial cells is performed for the number of CC3+ signals per 100 μm of the coelomic blood vessel. *n* = 3–4; sections counted = 6–8. Mean ± SEM. Unpaired Student’s t*-*test. *p < 0.05.

Given that TRPC3 is also expressed in endothelial cells and inhibition of vascularization disrupts testis cord formation and partitioning (Bott et al., 2006; Combes et al., 2009; Cool et al., 2011), we examined the potential non-Sertoli cell effects of Pyr3 treatment on vascularization using the endothelial cell marker PECAM1. At the 48-h time point, both control and Pyr3-treated XY gonads exhibited the formation of a typical coelomic blood vessel and regular vessels between testis cords (Figure 3A). This observation suggests that TRPC3 inhibition did not disrupt vascularization at this early stage, indicating that the reduction in germ cell numbers was unlikely due to vascular defects. However, after 72 h of culture, the coelomic vessel was disrupted in Pyr3-treated gonads when compared to control gonads (Figure 3A). This disruption suggests an altered vascularization pattern associated with TRPC3 inhibition during the later stages of culture. Subsequent analysis of endothelial cell apoptosis at the 48-h time point revealed that Pyr3-treated gonads showed a 4-fold increase in the number of cleaved Caspase-3 signals at the coelomic blood vessel when compared to control gonads (Figures 3B, C). These findings suggest that, although the coelomic blood vessel was not disturbed at the 48-h time point, it was already manifesting with increased endothelial cell apoptosis.

### 3.4 TRPC3 stimulates proliferation and controls cell morphology in NT2/D1 cells

**FIGURE 4:**
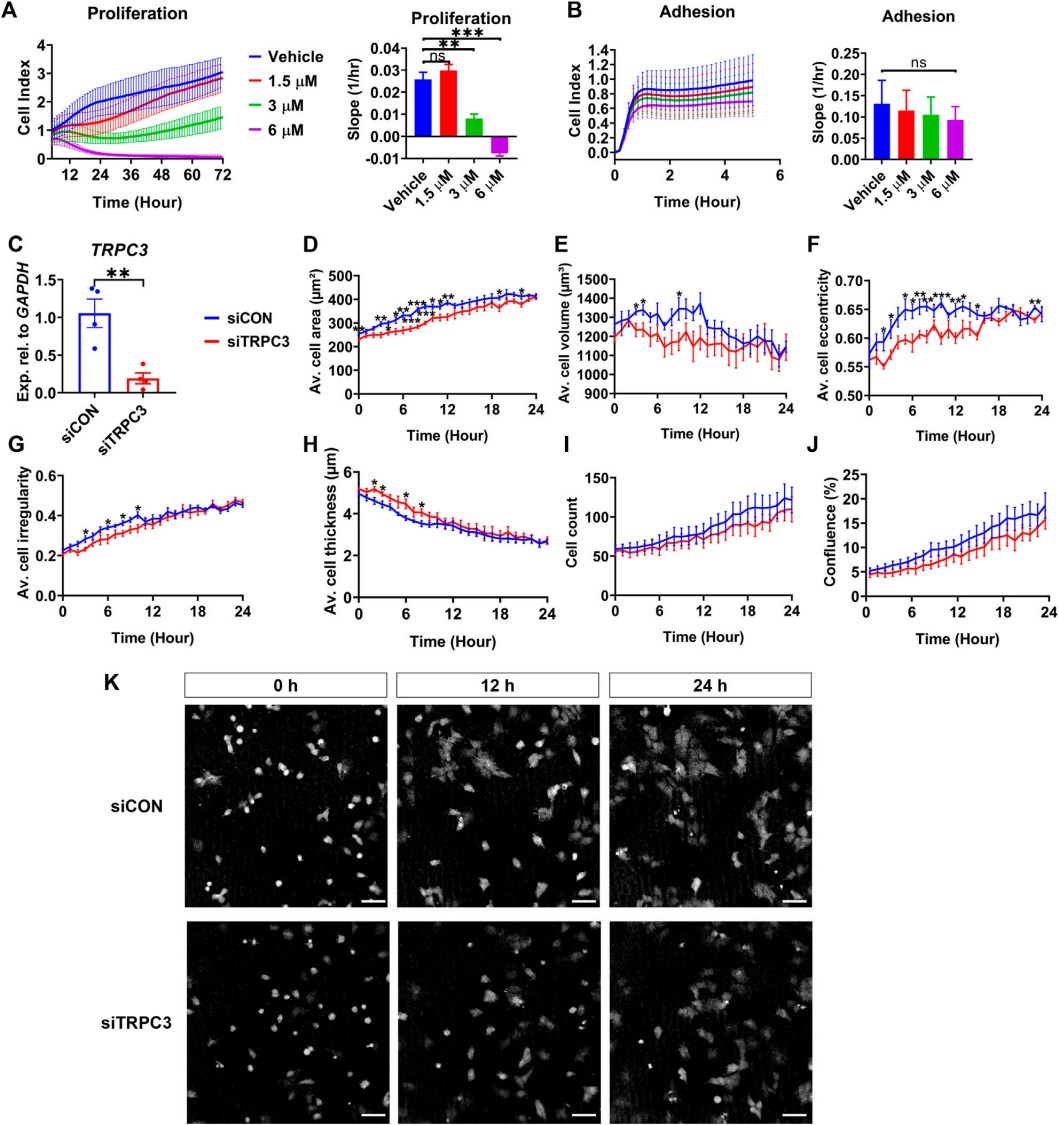
TRPC3 stimulates proliferation and controls cell morphology in NT2/D1 cells. **(A, B)** Cell proliferation (5–72 h) and cell adhesion (0–5 h) curves were generated from the xCELLigence system. Electrical impedance was measured and reported as cell index. The rate of cell proliferation or adhesion was calculated as the slope (1/hr). Mean ± SEM values were taken from three independent biological experiments. One-way ANOVA test. **p < 0.01, ***p < 0.001; ns, not significant. **(C)** qRT-PCR analysis showing the expression levels of *TRPC3* in control (siCON) and siTRPC3 groups. Data are presented as Mean ± SEM, with statistical significance determined by unpaired Student’s t*-*test. **p < 0.01, n = 4. **(D–J)** HoloMonitor measurements of cell morphology include cell area, volume, eccentricity, irregularity, thickness, count, and confluence. Mean ± SEM values represent the average of two independent experiments. Unpaired Student’s t*-*test. *p < 0.05, **p < 0.01, ***p < 0.001. **(K)** Holographic phase images depict cells from siCON and siTRPC3 groups at 0, 12 and 24 h. Scale bar = 50 μm.

To explore the impact of TRPC3 on Sertoli cell proliferation, we treated the human testicular cell line NT2/D1 with TRPC3 inhibitor Pyr3 at various concentrations, monitoring cell adhesion and proliferation in real-time over 72 h using the xCELLigence system. TRPC3 inhibition induced a dose-dependent reduction in cell proliferation, while cell adhesion remained unaffected (Figures 4A, B). To examine whether TRPC3 also regulates cell morphology over time, we silenced *TRPC3* in NT2/D1 cells using siRNA (Figure 4C) and measured cell morphology using the digital holographic microscope HoloMonitor. *TRPC3* knockdown resulted in a decrease cell area (Figure 4D), volume (Figure 4E), eccentricity (Figure 4F) and irregularity (Figure 4G), and an increase in cell thickness (Figure 4H), especially in the first 6–12 h. Both control (siCON) and *TRPC3* knockdown (siTRPC3) groups had similar initial densities with consistent increases over time, as indicated by the cell count and confluence resu*l*ts (Figures 4I, K). The *s*iTRPC3 group showed slower cell proliferation but no significant difference compared to the control group, consistent with xCELLigence results. These findings suggest that TRPC3 stimulates Sertoli cell proliferation and controls cell morphology *in vitro*.

## 4 Discussion

SOX9 is a critical transcription factor in testis development, and its downstream target genes, such as *Amh*, *Dhh*, and *Cyp26b1*, play crucial roles in this process (Ming et al., 2022). We hypothesized that potential SOX9 target genes are also significant contributors to testis formation and may represent potential candidates for genetic mutations associated with human Differences/Disorders of Sex Development (DSD). In this study, we investigated the function of *Trpc3*, a putative direct target gene of SOX9, in testis development. The *Trpc3* gene is expressed in Sertoli cells from E11.5 onwards, and we found that TRPC3 protein exhibited high expression levels in Sertoli cells from at least E13.5, suggesting its involvement in early testis development. Our *ex vivo* gonad culture experiments revealed its role in supporting male germ cell development. The effects were absent in female gonads, possibly attributed to the lack of *TRPC3* expression in female granulosa or stromal cells, highlighting the sex-specific effects of TRPC3. In addition, we demonstrated that *Trpc3*/TRPC3 gene and protein are also expressed in endothelial cells. Our *ex vivo* gonad culture experiments showed that TRPC3 is required to maintain the coelomic blood vessel. Overall, our findings indicate that TRPC3 has two distinct functions in XY gonads: in Sertoli cells to stimulate germ cell proliferation and in endothelial cells to ensure their survival.

We observed a significant downregulation of *Trpc3* expression in E13.5 Sox9 KO XY gonads, showing that *Trpc3* expression is largely dependent on SOX9. Published scRNA-seq data indicate *Trpc3* expression in fetal Sertoli cells (Supplementary Figure S1); we found that expression of *Trpc3* in Sertoli cells increases within developing male gonads after sex determination. This implies the existence of a sex-specific mechanism in XY gonads that upregulates Trpc3 expression during sex differentiation. Our previous SOX9 ChIP-seq data demonstrated SOX9 binding to the proximal promoter region and intron 1 of both mouse and bovine fetal testes (Supplementary Figure S6A, B) (Rahmoun et al., 2017). DNase I hypersensitivity data from human fetal testes and fetal ovaries shows increased chromatin accessibility in this region (Supplementary Figure S6B) (Kundaje et al., 2015), and ENCODE histone modifications in NT2/D1 cells indicates active regulatory potential (Supplementary Figure S6B) (ENCODE Project Consortium, 2012). Moreover, prediction of transcription factor binding sites within the SOX9 ChIP-seq peaks revealed three potential SOX9 binding sites and one GATA4 binding site in the Trpc3/TRPC3 promoter region (Supplementary Figure S6C). Based on these data, it appears likely that SOX9 directly binds to the proximal promoter and/or intron 1 of *Trpc3*/*TRPC3* to regulate its transcription in Sertoli cells. Following the initiation of *Trpc3*/*TRPC3* expression by SOX9, other transcription factors in Sertoli cells, such as SOX8, SOX10, and GATA4, may upregulate *Trpc3*/*TRPC3* expression cooperatively (Barrionuevo and Scherer, 2010; Sekido and Lovell-Badge, 2013; Eggers et al., 2014; Viger et al., 2022). Future investigations could utilize ChIP-qPCR and luciferase assays to validate these hypotheses.

The results from our *ex vivo* gonad culture experiments, in which we treated male gonads with the TRPC3 inhibitor Pyr3, provided valuable insights into the role of *Trpc3* in the developing testis. By 48 and 72 h, Pyr3-treated XY gonads showed a reduced number of germ cells due to decreased germ cell proliferation, alongside disrupted coelomic blood vessel and increased endothelial cell apoptosis. These results suggest a potential role for TRPC3 in male fertility. One *Trpc3* KO model, the Δ202 mice, disrupted the *Trpc3* promoter due to the SV40 T antigen transgene insertion, leading to the inhibition of transcription and subsequent knockout of the TRPC3 protein. This *Trpc3* KO mouse model presented severe phenotypes, including reduced average litter size, hindlimb atrophy, and progressive paralysis, ultimately leading to the death of these mice within 3–4 months (López-Revilla et al., 2004; Rodríguez-Santiago et al., 2007). The decreased average litter size in KO mice may be a result of decreased fertility and embryo viability. This *Trpc3* KO mouse model as well as our *ex vivo* gonad culture model could potentially serve as valuable tools for future investigations into TRPC3 roles in fetal testis development. In contrast, two types of whole-body *Trpc3* KO mouse models have been reported to be viable, fertile, and phenotypically normal compared to wildtype mice (Hartmann et al., 2008; Dong et al., 2017). These *Trpc3* KO mouse models were generated through the excision of either exon 7 or exons 7 and 8 from the *Trpc3* gene using a Cre-loxP-based strategy. Consequently, the *Trpc3* gene in these KO mice is expected to encode a truncated TRPC3 protein, which might retain some functional activity (Trebak, 2010).

Previously, a rare *TRPC3* variant (NM_001130698.2:c.2285G > A; p.Arg762His) (R762H) was identified in a patient with spinocerebellar ataxia type 41 (MIM#616410) (Fogel et al., 2015). The TRPC3 mutation falls within the highly conserved TRP box (EWKFAR) which is implicated in channel gating (Clapham, 2003; Fogel et al., 2015; Chen et al., 2021), and overexpression of the mutant TRPC3 protein led to significant cell death and increased nuclear localization of the calcium-responsive transcription factor NFAT (Fogel et al., 2015). We previously identified the same *TRPC3* R762H variant as one of only 24 candidate modifiers in a 46, XY DSD patient with an *FGF9* variant (c.583G > A; p. Asp195Asn) (Croft et al., 2023). To assess the pathogenicity of the *TRPC3* variant, knock-in mice could be generated and be crossed with *Fgf9* mutant mice to model the 46, XY DSD patient and assess the effects of both variants on testis development in mice. To test whether the mutant TRPC3 protein enhances channel activity and induces cell death, introducing *TRPC3* mutations into human cell lines such as NT2/D1 and newly generated human testis and ovary cell lines from induced pluripotent stem cells (iPSC) could provide valuable insights (Knarston et al., 2020; Gonen et al., 2023; Pierson Smela et al., 2023).

In summary, our study unveiled *Trpc3* as a novel direct target gene of SOX9, prominently expressed in Sertoli cells. Our *ex vivo* gonad culture experiments shed light on the potential role of TRPC3 in Sertoli, germ and endothelial cell development. Additionally, the *TRPC3* variant (c.2285G > A; p.Arg762His) may implicate a potential involvement of *TRPC3* in human DSD.

## Data Availability

The datasets presented in this study can be found in online repositories. The names of the repository/repositories and accession number(s) can be found in the article/Supplementary Material.

## Supplementary material

SUPPLEMENTARY FIGURE S1 UMAP (uniform manifold approximation and projection) of the cell lineages (color) in the male or female mouse gonad at ages E10.5, E11.5 and E12.5 (pooled data) scRNA-seq dataset from Garcia-Alonso et al. (Garcia-Alonso et al., 2022). In male: the Sertoli cell (chartreuse) and endothelial cell (green) clusters have been identified; in female: the pre-granulosa cell (pink) and endothelial cell (green) clusters have been identified. UMAP of gene expression shows *Sox9, Amh, Pecam1, Foxl2* and *Trpc3* gene expression across all cell lineages for either male or female embryonic mouse gonad. UMAP of stage indicates the embryonic day (E) of each derived cell within lineage clusters.

SUPPLEMENTARY FIGURE S2 Co-immunofluorescence staining of TRPC3 (green) with the endothelial cell and germ cell marker PECAM1 (red) in the mouse testis from E11.5 to E15.5, and with the Leydig cell marker HSD3B (red) at E14.5 and E15.5. Dashed white arrows indicate possible colocalization of TRPC3 and PECAM1. Arrowheads indicate HSD3B staining. Nuclei are visualized with the nuclear marker DAPI (blue). Scale bar = 50 µm.

SUPPLEMENTARY FIGURE S3 TRPC3 inhibition has no impact on cultured XX mouse gonads. **(A)** Immunofluorescence staining of female gonads after exposure to either the vehicle control DMSO or the TRPC3 inhibitor Pyr3 during ex vivo culture for 24, 48, and 72 h. The sections are stained for germ cell marker DDX4 (red) and somatic cell marker GATA4 (green). Nuclei are visualized with the nuclear marker DAPI (blue). Dashed lines outline gonads. Scale bar = 50 µm. **(B)** Quantification of germ cells in XX DMSO and XX Pyr3-treated gonads at 24, 48 and 72 h post-culture. n = 2; sections counted = 4–10. Mean ± SEM. Unpaired Student's t-test. ns, not significant.

SUPPLEMENTARY FIGURE S4 TRPC3 inhibition does not affect germ cell apoptosis in cultured XY gonads. **(A)** Apoptosis analysis at 24, 48, and 72 h in XY gonads treated with DMSO or Pyr3 by immunofluorescence staining for the cell apoptosis marker cleaved Caspase-3 (CC3) (green) and the germ cell marker DDX4 (red). Arrowheads indicate examples of apoptotic germ cells. Nuclei are visualized with the nuclear marker DAPI (blue). Scale bar = 50 μm. Dashed lines outline gonads. **(B)** Quantification of apoptotic germ cells is performed for CC3+ germ cells relative to the total germ cell population. n = 2–4; sections counted = 5–11. Data are represented as Mean ± SEM. Unpaired Student's t-test. ns, not significant.

SUPPLEMENTARY FIGURE S5 TRPC3 inhibition has no impact on testis cord formation in cultured XY gonads. Immunofluorescence staining of Laminin (green) and GATA4 (red) in control (XY DMSO) and Pyr3-treated (XY Pyr3) gonads cultured *ex vivo* for 24, 48 and 72 h. Nuclei are visualized with the nuclear marker DAPI (blue). Yellow dashed lines outline gonads. White dashed lines outline testis cords. Scale bar = 50 μm.

SUPPLEMENTARY FIGURE S6 Analysis of SOX9 ChIP peaks in the *Trpc3/TRPC3* gene. **(A)** SOX9 ChIP-seq peaks indicate regions bound by SOX9 within the *Trpc3* gene in mouse fetal testes (Rahmoun et al., 2017), visualized using IGV. **(B)** The schematic presents key regulatory features of the *Trpc3/ TRPC3* gene visualized on the UCSC genome browser. Black boxes indicate the locations of SOX9 ChIP peaks within the *Trpc3/TRPC3* gene in mouse and bovine fetal testes (Rahmoun et al., 2017). Bioinformatic tracks from the UCSC genome browser include the DNase I hypersensitivity data from human fetal testis and ovary (Kundaje et al., 2015), the 100-vertebrate conservation track, and ENCODE histone modification peaks present in the human testicular cell line NT2/D1 (ENCODE Project Consortium, 2012). **(C)** Alignment of the *Trpc3/TRPC3* promoter regions of mouse, bovine and human. Potential SOX9 and GATA4 binding motifs were predicted by PROMO (Farré et al., 2003) and JASPAR (Rauluseviciute et al., 2024) programs. The potential binding motifs for SOX9 and GATA4 are highlighted in red and green, respectively. Nucleotide conservation between mouse, bovine and human is indicated by asterisks (*). The transcription start site (TSS) is marked with an angled arrow.
