# Corrigendum: A role for TRPC3 in mammalian testis development

**DOI:** 10.3389/fcell.2024.1484634

**Published:** 2024-10-15

**Authors:** Zhenhua Ming, Stefan Bagheri-Fam, Emily R. Frost, Janelle M. Ryan, Brittany Vining, Vincent R. Harley

**Affiliations:** ^1^ Sex Development Laboratory, Hudson Institute of Medical Research, Melbourne, VIC, Australia; ^2^ Department of Molecular and Translational Science, Monash University, Melbourne, VIC, Australia

**Keywords:** SOX9, testis, sertoli cells, DSD, TRPC3, TRP, sex determination

In the published article, there was an error in the **Author list**, and author **Brittany Vining** was erroneously excluded. The corrected author list appears below.

“Zhenhua Ming^1,2^, Stefan Bagheri-Fam^1^, Emily R. Frost^1^, Janelle M. Ryan^1,2^, Brittany Vining^1,2^ and Vincent R. Harley^1,2^”

The corrected **Author contributions** statement appears below.

“ZM: Conceptualization, Formal Analysis, Investigation, Methodology, Project administration, Visualization, Writing–original draft, Writing–review and editing. SB-F: Conceptualization, Data curation, Software, Supervision, Writing–review and editing. EF: Supervision, Writing–review and editing. JR: Methodology, Writing–review and editing. BV: Visualization, Writing–review and editing. VH: Conceptualization, Funding acquisition, Project administration, Resources, Supervision, Writing–review and editing.”

In the published article, **Supplementary Figure 1** was mistakenly not included in the publication. The missing material appears below.

**Figure:**
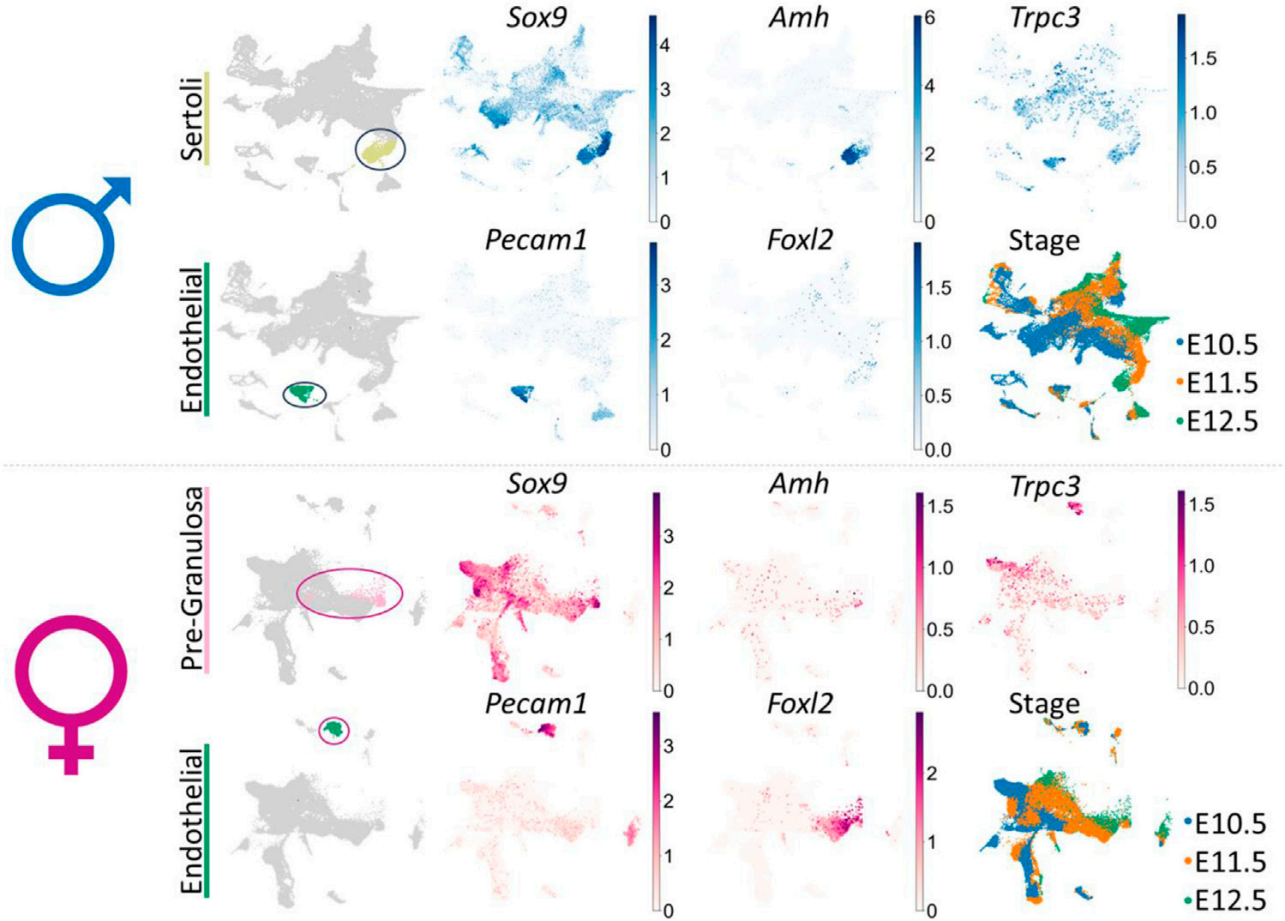
**SUPPLEMENTARY FIGURE S1** UMAP (uniform manifold approximation and projection) of the cell lineages (color) in the male or female mouse gonad at ages E10.5, E11.5 and E12.5 (pooled data) scRNA-seq dataset from Garcia-Alonso et al. (**Garcia-Alonso et al., 2022**). In male: the Sertoli cell (chartreuse) and endothelial cell (green) clusters have been identified; in female: the pre-granulosa cell (pink) and endothelial cell (green) clusters have been identified. UMAP of gene expression shows *Sox9, Amh, Pecam1, Foxl2* and *Trpc3* gene expression across all cell lineages for either male or female embryonic mouse gonad. UMAP of stage indicates the embryonic day (E) of each derived cell within lineage clusters.

In the published article, the legends of the **Supplementary Figures** were mistakenly not included in the publication. The missing material appears below.

“**SUPPLEMENTARY FIGURE S1** UMAP (uniform manifold approximation and projection) of the cell lineages (color) in the male or female mouse gonad at ages E10.5, E11.5 and E12.5 (pooled data) scRNA-seq dataset from Garcia-Alonso et al. (**Garcia-Alonso et al., 2022**). In male: the Sertoli cell (chartreuse) and endothelial cell (green) clusters have been identified; in female: the pre-granulosa cell (pink) and endothelial cell (green) clusters have been identified. UMAP of gene expression shows *Sox9, Amh, Pecam1, Foxl2* and *Trpc3* gene expression across all cell lineages for either male or female embryonic mouse gonad. UMAP of stage indicates the embryonic day (E) of each derived cell within lineage clusters.

**SUPPLEMENTARY FIGURE S2** Co-immunofluorescence staining of TRPC3 (green) with the endothelial cell and germ cell marker PECAM1 (red) in the mouse testis from E11.5 to E15.5, and with the Leydig cell marker HSD3B (red) at E14.5 and E15.5. Dashed white arrows indicate possible colocalization of TRPC3 and PECAM1. Arrowheads indicate HSD3B staining. Nuclei are visualized with the nuclear marker DAPI (blue). Scale bar = 50 μm.

**SUPPLEMENTARY FIGURE S3** TRPC3 inhibition has no impact on cultured XX mouse gonads. **(A)** Immunofluorescence staining of female gonads after exposure to either the vehicle control DMSO or the TRPC3 inhibitor Pyr3 during *ex vivo* culture for 24, 48, and 72 h. The sections are stained for germ cell marker DDX4 (red) and somatic cell marker GATA4 (green). Nuclei are visualized with the nuclear marker DAPI (blue). Dashed lines outline gonads. Scale bar = 50 μm. **(B)** Quantification of germ cells in XX DMSO and XX Pyr3-treated gonads at 24, 48 and 72 h post-culture. n = 2; sections counted = 4–10. Mean ± SEM. Unpaired Student’s t-test. ns, not significant.

**SUPPLEMENTARY FIGURE S4** TRPC3 inhibition does not affect germ cell apoptosis in cultured XY gonads. **(A)** Apoptosis analysis at 24, 48, and 72 h in XY gonads treated with DMSO or Pyr3 by immunofluorescence staining for the cell apoptosis marker cleaved Caspase-3 (CC3) (green) and the germ cell marker DDX4 (red). Arrowheads indicate examples of apoptotic germ cells. Nuclei are visualized with the nuclear marker DAPI (blue). Scale bar = 50 μm. Dashed lines outline gonads. **(B)** Quantification of apoptotic germ cells is performed for CC3+ germ cells relative to the total germ cell population. n = 2–4; sections counted = 5–11. Data are represented as Mean ± SEM. Unpaired Student’s t-test. ns, not significant.

**SUPPLEMENTARY FIGURE S5** TRPC3 inhibition has no impact on testis cord formation in cultured XY gonads. Immunofluorescence staining of Laminin (green) and GATA4 (red) in control (XY DMSO) and Pyr3-treated (XY Pyr3) gonads cultured *ex vivo* for 24, 48 and 72 h. Nuclei are visualized with the nuclear marker DAPI (blue). Yellow dashed lines outline gonads. White dashed lines outline testis cords. Scale bar = 50 μm.

**SUPPLEMENTARY FIGURE S6** Analysis of SOX9 ChIP peaks in the *Trpc3/TRPC3* gene. **(A)** SOX9 ChIP-seq peaks indicate regions bound by SOX9 within the *Trpc3* gene in mouse fetal testes (**Rahmoun et al., 2017**), visualized using IGV. **(B)** The schematic presents key regulatory features of the *Trpc3/TRPC3* gene visualized on the UCSC genome browser. Black boxes indicate the locations of SOX9 ChIP peaks within the *Trpc3/TRPC3* gene in mouse and bovine fetal testes (**Rahmoun et al., 2017**). Bioinformatic tracks from the UCSC genome browser include the DNase I hypersensitivity data from human fetal testis and ovary (**Kundaje et al., 2015**), the 100-vertebrate conservation track, and ENCODE histone modification peaks present in the human testicular cell line NT2/D1 (**ENCODE Project Consortium, 2012**). **(C)** Alignment of the *Trpc3/TRPC3* promoter regions of mouse, bovine and human. Potential SOX9 and GATA4 binding motifs were predicted by PROMO (**Farré et al., 2003**) and JASPAR (**Rauluseviciute et al., 2024**) programs. The potential binding motifs for SOX9 and GATA4 are highlighted in red and green, respectively. Nucleotide conservation between mouse, bovine and human is indicated by asterisks (*). The transcription start site (TSS) is marked with an angled arrow.”

In the published article, there was an error in **Supplementary Figure 5**. The incorrect image was published, and the numbering of the figure and its in-text citations were not accurate. The correct material appears below and is now correctly labelled as **Supplementary Figure 6**.

**Figure:**
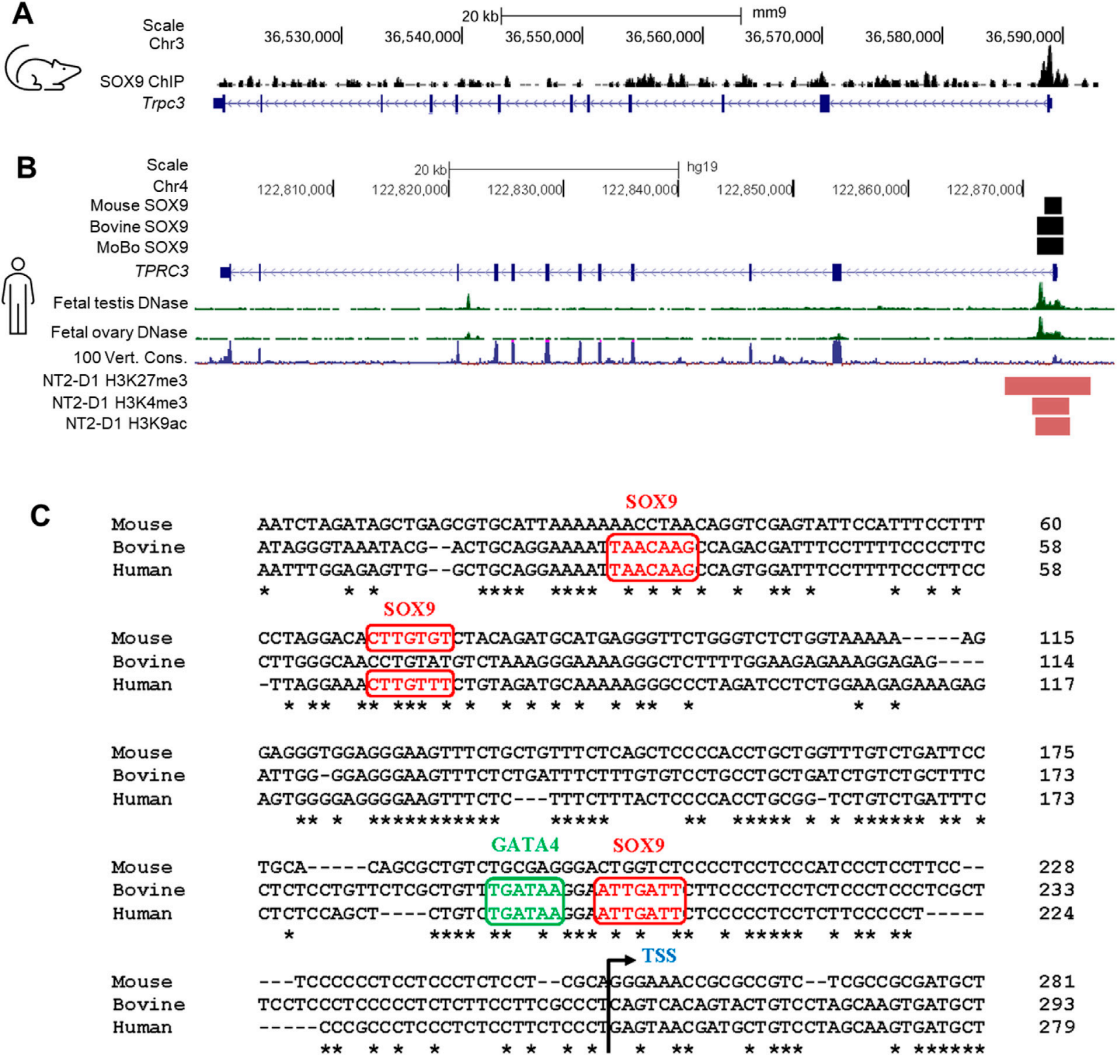
**SUPPLEMENTARY FIGURE S6** Analysis of SOX9 ChIP peaks in the *Trpc3/TRPC3* gene. **(A)** SOX9 ChIP-seq peaks indicate regions bound by SOX9 within the *Trpc3* gene in mouse fetal testes (**Rahmoun et al., 2017**), visualized using IGV. **(B)** The schematic presents key regulatory features of the Trpc3/TRPC3 gene visualized on the UCSC genome browser. Black boxes indicate the locations of SOX9 ChIP peaks within the *Trpc3/TRPC3* gene in mouse and bovine fetal testes (**Rahmoun et al., 2017**). Bioinformatic tracks from the UCSC genome browser include the DNase I hypersensitivity data from human fetal testis and ovary (**Kundaje et al., 2015**), the 100-vertebrate conservation track, and ENCODE histone modification peaks present in the human testicular cell line NT2/D1 (**ENCODE Project Consortium, 2012**). **(C)** Alignment of the *Trpc3/TRPC3* promoter regions of mouse, bovine and human. Potential SOX9 and GATA4 binding motifs were predicted by PROMO (**Farré et al., 2003**) and JASPAR (**Rauluseviciute et al., 2024**) programs. The potential binding motifs for SOX9 and GATA4 are highlighted in red and green, respectively. Nucleotide conservation between mouse, bovine and human is indicated by asterisks (*). The transcription start site (TSS) is marked with an angled arrow.

In the published article, there was an error. The correct **Supplementary information** was not included, and the in-text citations of some **Supplementary Figures** were not correct.

A correction has been made to **Results**, *TRPC3 is strongly expressed in Sertoli cells from E13.5*, Paragraph 1. This sentence previously stated:

“We further investigated *Trpc3* expression during testis development by screening previous microarray data of male and female developing gonads from E11.5 to E13.5 (**Jameson et al., 2012**).”

The corrected sentence appears below:

“We further investigated *Trpc3* expression during testis development by screening previous microarray data of male and female developing gonads from E11.5 to E13.5 (**Jameson et al., 2012**) and querying expression of *Trpc3* in isolated cell lineages of the developing male or female mouse gonad (**Supplementary Figure 1**).”

A correction has been made to **Results**, *TRPC3 is strongly expressed in Sertoli cells from E13.5*, Paragraph 2. These sentences previously stated:

“At E12.5, E13.5 and E15.5, weak TRPC3 staining was found in a subset of endothelial cells of the coelomic blood vessel (**Supplementary Figure S1**). From E14.5 onwards, weak TRPC3 staining was also detectable in interstitial cells (**Figures 1C, D**). Co-immunostaining for TRPC3 and the Leydig cell marker HSD3B revealed that these are Leydig cells (**Supplementary Figure S1**). However, since these signals were also detected in the PECAM1 channel, it is possible that they represent nonspecific background staining (**Supplementary Figure S1**).”

The corrected sentences appear below:

“At E12.5, E13.5 and E15.5, weak TRPC3 staining was found in a subset of endothelial cells of the coelomic blood vessel (**Supplementary Figure S2**). From E14.5 onwards, weak TRPC3 staining was also detectable in interstitial cells (**Figures 1C, D**). Co-immunostaining for TRPC3 and the Leydig cell marker HSD3B revealed that these are Leydig cells (**Supplementary Figure S2**). However, since these signals were also detected in the PECAM1 channel, it is possible that they represent nonspecific background staining (**Supplementary Figure S2**).”

A correction has been made to **Results**, *Inhibition of TRPC3 leads to a reduction in germ cell numbers in cultured XY gonads*, Paragraph 1. This sentence previously stated:

“Distinct from the male gonads, TRPC3 inhibition did not cause any changes in germ cell numbers in female gonads (**Supplementary Figure S2**), highlighting a sex-specific response to TRPC3 inhibition.”

The corrected sentence appears below:

“Distinct from the male gonads, TRPC3 inhibition did not cause any changes in germ cell numbers in female gonads (**Supplementary Figure S3**), highlighting a sex-specific response to TRPC3 inhibition.”

A correction has been made to **Results**, *Inhibition of TRPC3 leads to a reduction in germ cell numbers in cultured XY gonads,* Paragraph 2. These sentences previously stated:

“Analysis of germ cell apoptosis revealed no significant differences between XY control gonads and XY Pyr3-treated gonads at the 24, 48 and 72-h time points (**Supplementary Figure S3**). Collectively, these findings suggest that the reduction of the germ cell population following TRPC3 inhibition is caused by decreased germ cell proliferation. Immunofluorescence analysis for Laminin, a marker for the basal lamina of testis cords, revealed that after 48 and 72 h of culture, both control and Pyr3-treated XY gonads had formed well-defined testis cords, wherein Laminin and a closely associated layer of Sertoli cells encircled both germ cells and Sertoli cells (**Supplementary Figure S4**).”

The corrected sentences appear below:

“Analysis of germ cell apoptosis revealed no significant differences between XY control gonads and XY Pyr3-treated gonads at the 24, 48 and 72-h time points (**Supplementary Figure S4**). Collectively, these findings suggest that the reduction of the germ cell population following TRPC3 inhibition is caused by decreased germ cell proliferation. Immunofluorescence analysis for Laminin, a marker for the basal lamina of testis cords, revealed that after 48 and 72 h of culture, both control and Pyr3-treated XY gonads had formed well-defined testis cords, wherein Laminin and a closely associated layer of Sertoli cells encircled both germ cells and Sertoli cells (**Supplementary Figure S5**).”

A correction has been made to **Discussion,** Paragraph 2. These sentences previously stated:

“We observed a significant downregulation of *Trpc3* expression in E13.5 *Sox9* KO XY gonads, showing that *Trpc3* expression is largely dependent on SOX9. We also found that expression of *Trpc3* in Sertoli cells increases within developing male gonads after sex determination. This implies the existence of a sex-specific mechanism in XY gonads that upregulates *Trpc3* expression during sex differentiation. Our previous SOX9 ChIP-seq data demonstrated SOX9 binding to the proximal promoter region and intron 1 of both mouse and bovine fetal testes (**Supplementary Figure S5A**) (**Rahmoun et al., 2017**). DNase I hypersensitivity data from human fetal testes and fetal ovaries shows increased chromatin accessibility in this region (**Supplementary Figure S5A**) (**Kundaje et al., 2015**), and ENCODE histone modifications in NT2/D1 cells indicates active regulatory potential (**Supplementary Figure S5A**) (**ENCODE Project Consortium, 2012**). Moreover, prediction of transcription factor binding sites within the SOX9 ChIP-seq peaks revealed three potential SOX9 binding sites and one GATA4 binding site in the *Trpc3*/*TRPC3* promoter region (**Supplementary Figure S5B**).”

The corrected sentences appear below:

“We observed a significant downregulation of *Trpc3* expression in E13.5 *Sox9* KO XY gonads, showing that *Trpc3* expression is largely dependent on SOX9. Published scRNA-seq data indicate *Trpc3* expression in fetal Sertoli cells (**Supplementary Figure 1**); we found that expression of *Trpc3* in Sertoli cells increases within developing male gonads after sex determination. This implies the existence of a sex-specific mechanism in XY gonads that upregulates Trpc3 expression during sex differentiation. Our previous SOX9 ChIP-seq data demonstrated SOX9 binding to the proximal promoter region and intron 1 of both mouse and bovine fetal testes (**Supplementary Figure S6A, B**) (**Rahmoun et al., 2017**). DNase I hypersensitivity data from human fetal testes and fetal ovaries shows increased chromatin accessibility in this region (**Supplementary Figure S6B**) (**Kundaje et al., 2015**), and ENCODE histone modifications in NT2/D1 cells indicates active regulatory potential (**Supplementary Figure S6B**) (**ENCODE Project Consortium, 2012**). Moreover, prediction of transcription factor binding sites within the SOX9 ChIP-seq peaks revealed three potential SOX9 binding sites and one GATA4 binding site in the *Trpc3/TRPC3* promoter region (**Supplementary Figure S6C**).”

The authors apologize for these errors and state that this does not change the scientific conclusions of the article in any way. The original article has been updated.

