# Supplementary figures and images for: A role for TRPC3 in mammalian testis development
Source: Front Cell Dev Biol. 2024 Feb 15;12:1337714. doi: 10.3389/fcell.2024.1337714 (PMC10902130; doi:10.3389/fcell.2024.1337714)

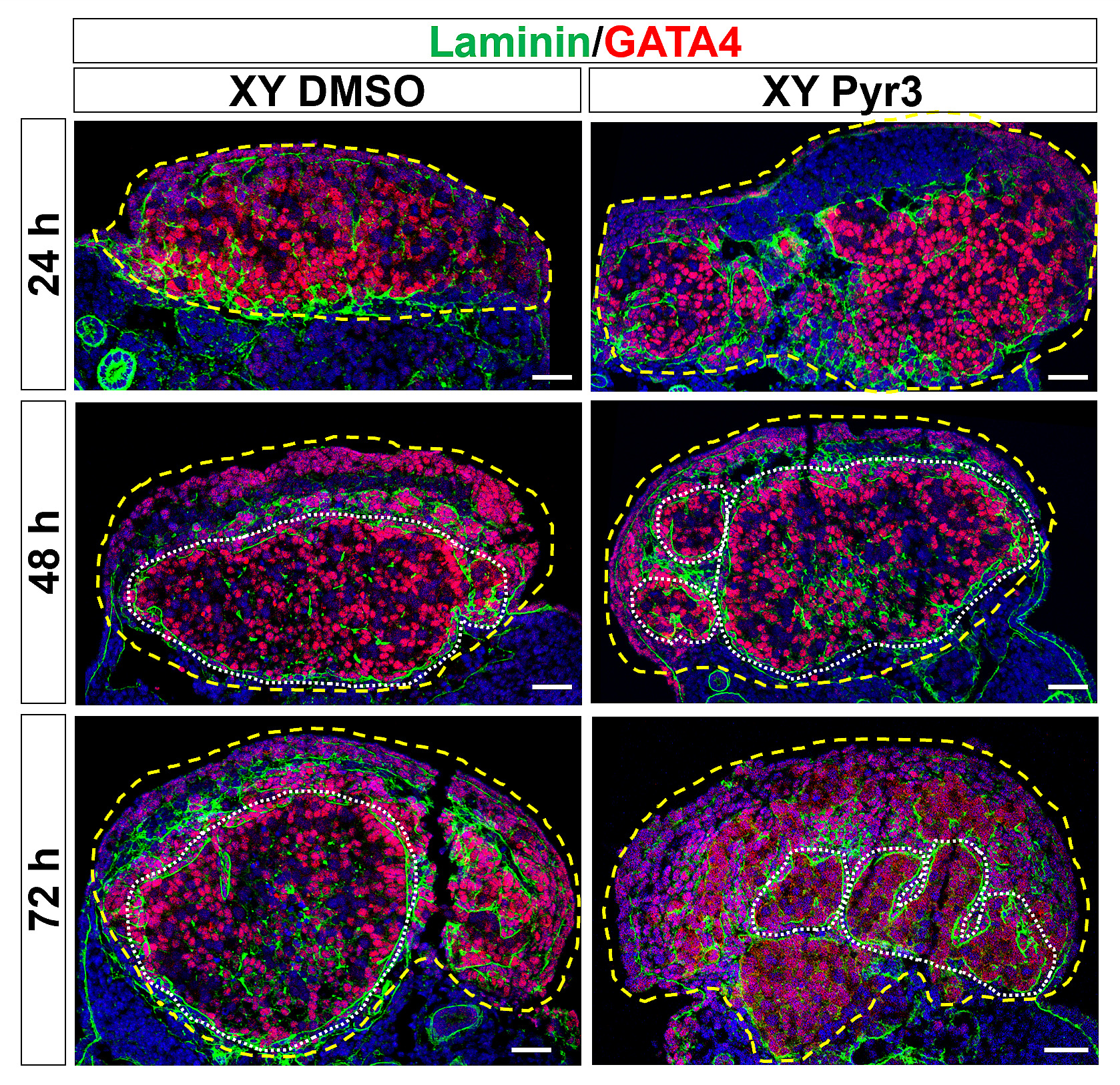

Supplement: Supplementary file 1 [file Image5.jpg]

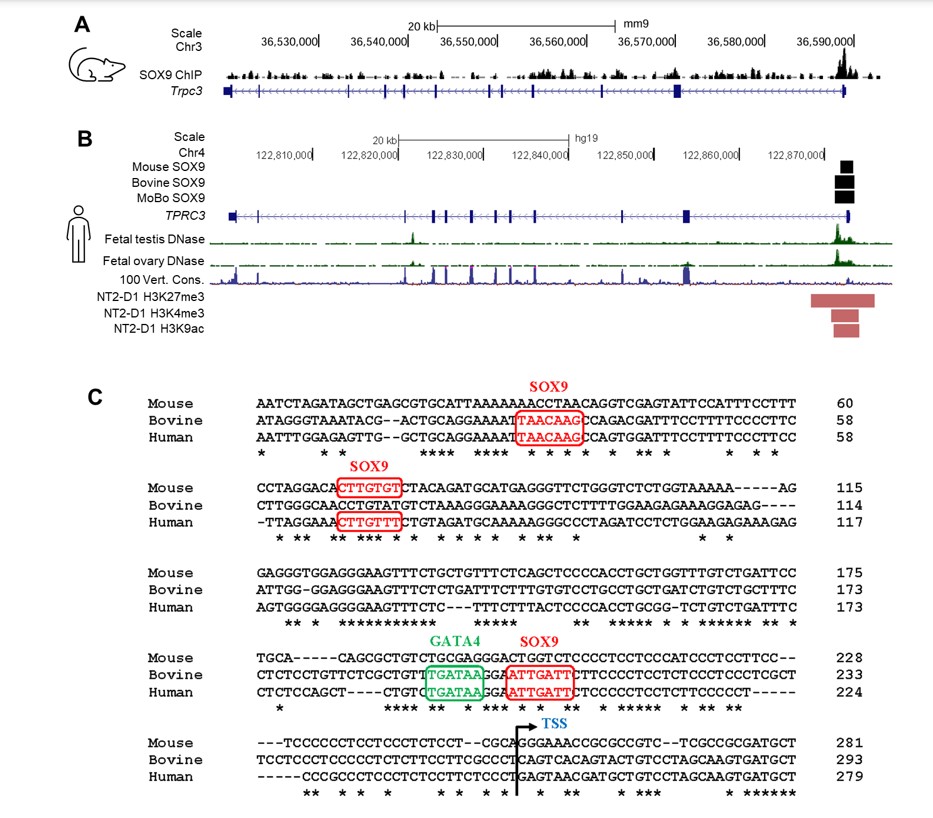

Supplement: Supplementary file 2 [file Image6.jpg]

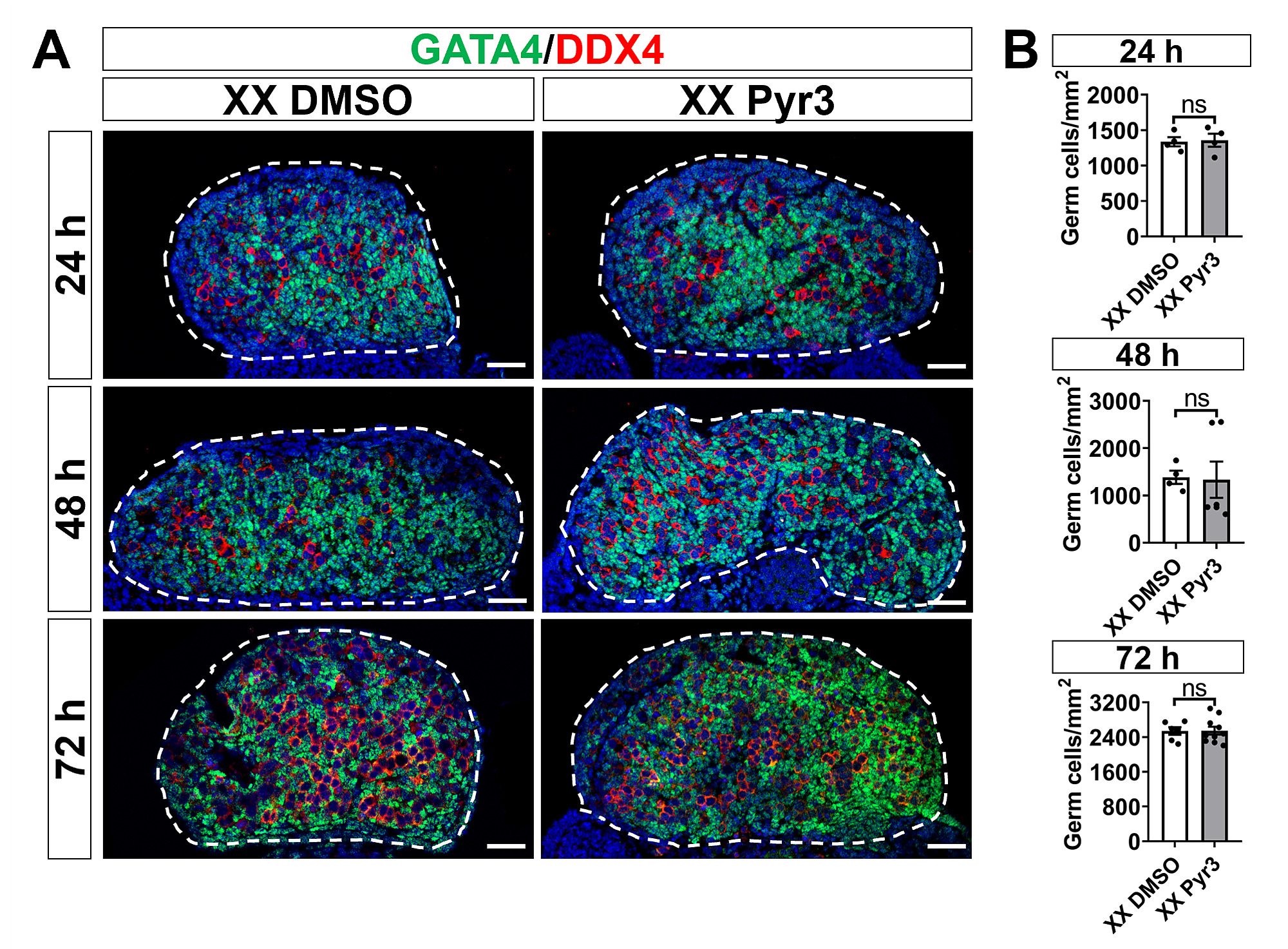

Supplement: Supplementary file 3 [file Image3.jpg]

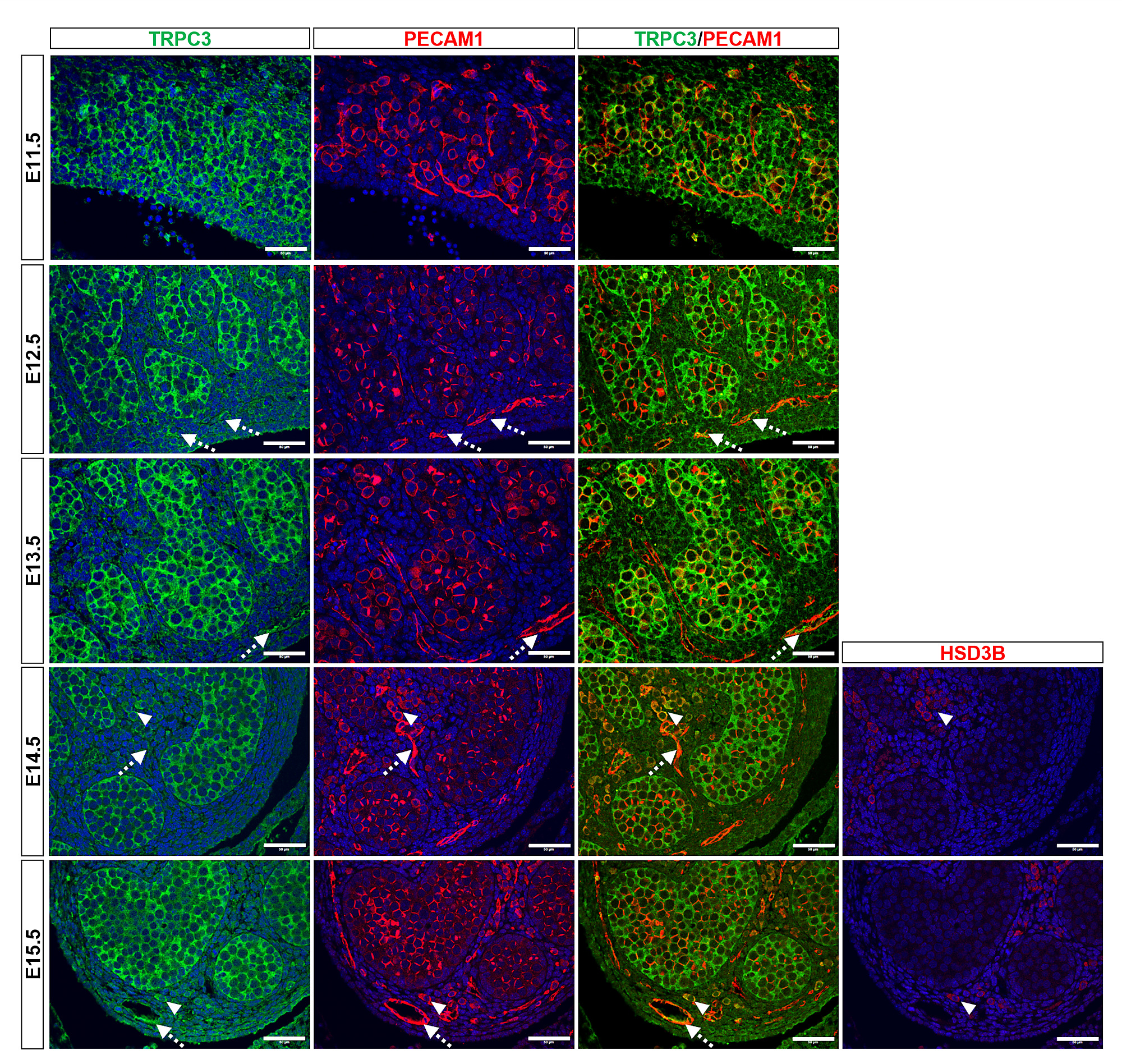

Supplement: Supplementary file 4 [file Image2.jpg]

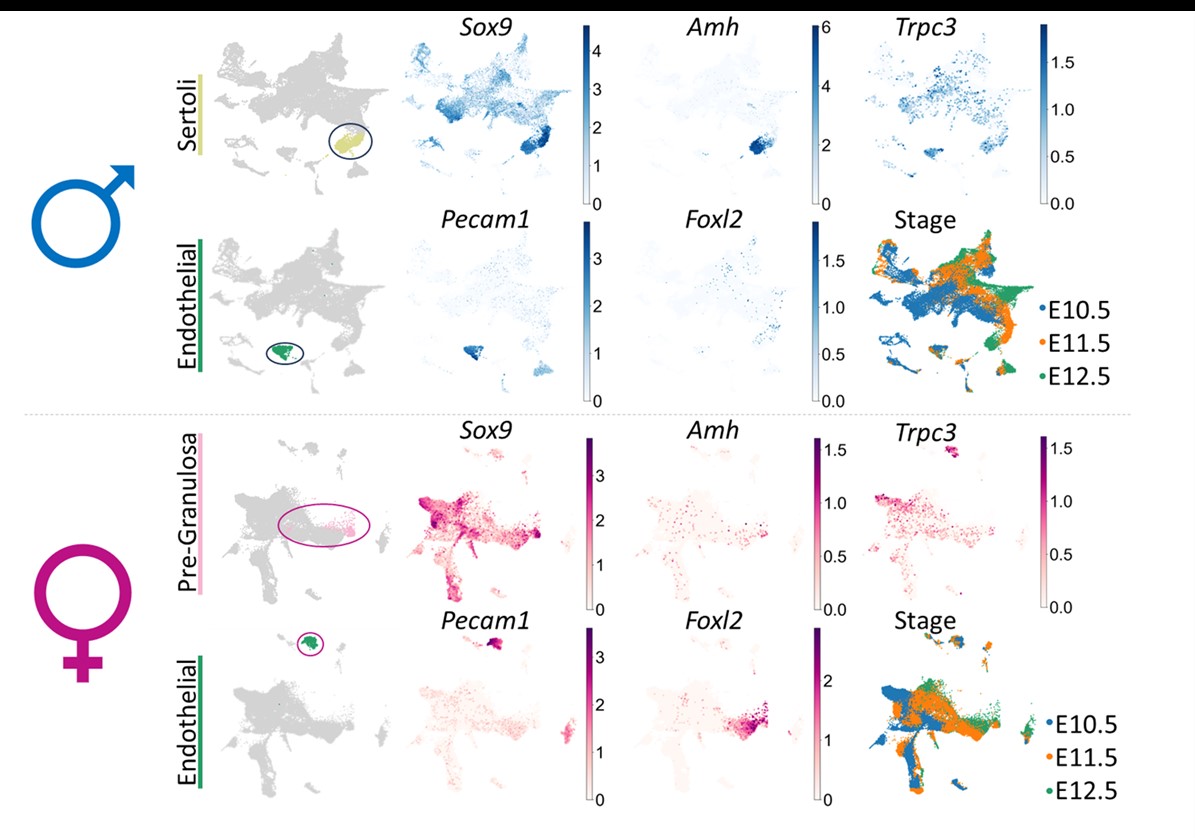

Supplement: Supplementary file 5 [file Image1.jpeg]

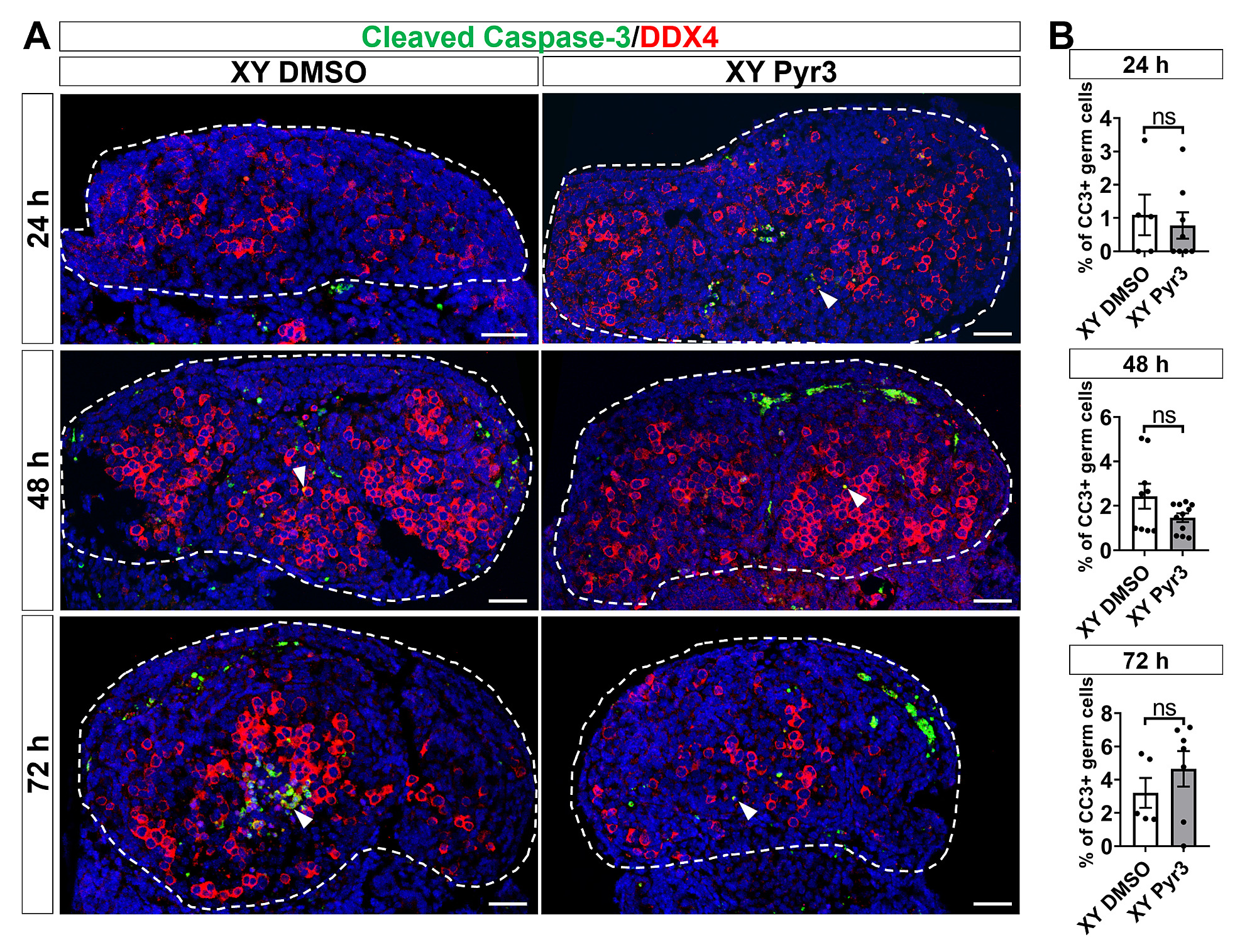

Supplement: Supplementary file 6 [file Image4.jpg]
